# The Combined Use of Triamcinolone and Platelet-Rich Plasma in Equine Metacarpophalangeal Joint Osteoarthritis Treatments: An In Vivo and In Vitro Study

**DOI:** 10.3390/ani14243645

**Published:** 2024-12-17

**Authors:** Kübra Guidoni, Elisabetta Chiaradia, Marco Pepe, Antonio Di Meo, Alessia Tognoloni, Matteo Seccaroni, Francesca Beccati

**Affiliations:** 1 Veterinary Teaching Hospital, Department of Veterinary Medicine, University of Perugia, Via San Costanzo 4, 06126 Perugia, Italy; vetkubra.d@gmail.com (K.G.); marco.pepe@unipg.it (M.P.); antonio.dimeo@unipg.it (A.D.M.); alessia.tognoloni@gmail.com (A.T.); matteo.seccaroni1@studenti.unipg.it (M.S.); francesca.beccati@unipg.it (F.B.); 2 Sport Horse Research Center, Department of Veterinary Medicine, University of Perugia, Via San Costanzo 4, 06126 Perugia, Italy

**Keywords:** osteoarthritis, equine, corticosteroids, metacarpophalangeal joint, platelet-rich plasma, intra-articular

## Abstract

Intra-articular corticosteroids, such as triamcinolone acetonide (TA), help reduce pain related to osteoarthritis (OA), but they may impair cartilage metabolism. In contrast, platelet-rich plasma (PRP) therapy, a regenerative therapy, has shown potential to promote healing and regeneration of articular cartilage. This study investigates the effects of combining PRP with TA to treat osteoarthritis in racehorses. The study proposes that PRP injection following TA treatment could reduce side effects and improve treatment outcomes. Firstly, in the in vitro study, chondrocytes were exposed to different TA concentrations, with or without PRP. TA dramatically reduced chondrocyte viability. However, this was prevented by the addition of PRP, which also increased cell proliferation. In the in vivo study, 32 racehorses with metacarpophalangeal (MCP) joint OA were separated into two groups: one received only TA, while the other received TA followed by PRP. For both groups, there were improved flexion assessments one week following the last treatment, but by two weeks following the last treatment, only TA+PRP had improved flexion assessments. TA+PRP also had improved lameness scores two weeks after the last treatment. In conclusion, combining PRP with TA could enhance chondrocyte viability and provide a better long-term therapeutic option for treating OA in racehorses. Further trials are required to thoroughly assess this technique’s safety and efficacy.

## Simple Summary

Osteoarthritis is a common joint condition in horses that is managed mainly with intraarticular treatments. While corticosteroids, such as triamcinolone acetonide, are still commonly used, regenerative treatments, such as platelet-rich plasma, have gained more attention in research studies. The aim of this study was to assess the efficiency of triamcinolone acetonide in combination with platelet-rich plasma in treating osteoarthritis in racehorses. The in vitro study demonstrated that platelet-rich plasma protects cartilage cells from the adverse effects of triamcinolone acetonide by enhancing cell viability. The in vivo study of 32 horses indicated that those treated with triamcinolone acetonide and platelet-rich plasma had better treatment outcomes than those treated with triamcinolone acetonide alone. The combination of these two treatments may improve clinical outcomes by reducing corticosteroid-induced adverse effects on cartilage cells.

## 1. Introduction

Osteoarthritis (OA) in horses is a chronic and degenerative condition with clinical manifestations such as synovitis, varying degrees of lameness, and a progressive loss of joint function. Among equine athletes, the metacarpophalangeal (MCP) joint emerges as a commonly affected joint and can develop both traumatic and degenerative lesions [1]. The impact of OA on the MCP joint holds significant implications for lameness in particular, resulting in substantial losses in training days and economic burden within the Thoroughbred racehorse industry [2].

In equine orthopedics, the routine intra-articular (IA) administration of corticosteroid as an initial treatment for osteoarthritis is based on the ability of corticosteroids to provide short-term symptomatic relief, provide potent anti-inflammatory, and improve joint mobility, reduce lameness and joint effusion in horses with synovitis and osteoarthritis [3,4]. Among various corticosteroids, triamcinolone acetonide (TA) is the most widely used due to its medium duration of action, which has been associated with beneficial effects on articular cartilage [3,5,6,7,8]. However, several studies have also identified potential detrimental effects of corticosteroids on articular cartilage composition and morphology [7,9,10]. While these negative effects are now known to be related to the type and dose of corticosteroid used, the frequency of repeated administration and joint loading after injection, it does imply IA corticosteroids should be used judiciously [3,8]. Results of in vitro and in vivo research indicate the use of betamethasone did not show any detrimental effects on articular cartilage, while the methylprednisolone acetonide had deleterious effects. The impact of TA is still debated as it seems to elicit both positive and detrimental effects in terms of equine cartilage metabolism [6,11,12]. However, as with all corticosteroids, the potential for unintentional alteration of cartilage metabolism is also present with TA [3].

Developing techniques for intra-articular therapies in equine athletes that increase tissue regeneration is critical, with PRP emerging as a potentially regenerative treatment [13]. PRP therapy is becoming more widely accepted [14,15,16]. PRP contains growth factors capable of stimulating tissue regeneration; these factors promote the proliferation and differentiation of chondrocytes and possess anti-inflammatory properties [17]. Additionally, PRP has been shown to protect chondrocytes from damage caused by various stressors and drugs [18,19,20]. Moreover, there is growing evidence to support its potential analgesic and anti-inflammatory properties, notably in the treatment of OA. PRP injections have been found to reduce pro-inflammatory cytokines and increase anti-inflammatory substances in the joint environment; in addition, this action helps to reduce the overall inflammatory response, which contributes to clinical signs relief in conditions of OA [14,15]. PRP is seen as a cost-effective and low-risk therapeutic option that uses the recipient’s own biological material to reduce the chance of adverse effects [16]. Several in vitro studies on chondrocytes and tenocytes have shown that corticosteroids have negative effects, while the addition of PRP to these medications significantly reduces cytotoxicity by modulating apoptosis and promoting cell proliferation [19,20,21]. The combination of TA and growth hormones also showed promise in improving anabolic metabolism in the articular cartilage [22]. Human clinical research examined in the literature reveals that PRP can fill cartilage defects, promote cartilage repair, alleviate OA symptoms, and improve joint function, all while maintaining an acceptable safety profile [23]. To the authors’ knowledge, very limited information exists regarding the impact of the combination of TA and PRP in equine orthopedics. With the extensive use of corticosteroid injections in equine practice, it is critical to fully understand their effects on the targeted tissues. While these injections have well-documented advantages, their related side effects highlight the need for new approaches to long-term joint treatment that try to mitigate potential negative consequences [24].

The aim of this study was to determine how the combination of TA and PRP might improve the clinical signs of MCP joint OA in racehorses. Authors hypothesized that the use of PRP after a single dose of TA may potentially improve the clinical signs of MCP joint OA longer than injection of TA alone.

## 2. Materials and Methods

### 2.1. In Vitro Study

#### 2.1.1. Primary Cultures of Equine Chondrocytes

The in vitro study was performed using chondrocytes isolated from metacarpo/metatarsophalangeal joints of six Thoroughbred horses 4.5 ± 1.3 years old submitted to euthanasia for reasons unrelated to this study; the quality of the biological material was not compromised by their clinical condition. The articular cartilage was harvested post-mortem from the weight-bearing surfaces of the metacarpo/metatarsophalangeal joints, according to Mancini et al. (2017) [25]. Equine tissues were used in accordance with the guidelines of the Animal Care and Use Committee of Perugia University. All the articular surfaces were exposed by making a careful incision around the metacarpo/metatarsophalangeal joint with a sterile scalpel and freeing it from the surrounding tissues. After macroscopic examination, the cartilage tissues showing structural integrity, consisting in no visible damage, such as tears, cracks, or breaks, and no signs of degenerative changes were collected using scalpel. Tissues were then washed three times in Dulbecco’s phosphate-buffered saline (PBS) without Ca^2+^ and Mg^2+^, containing penicillin (100 U/mL), streptomycin (100 mg/mL), and amphotericin B (250 μg/mL) (EuroClone, Milan, Italy) and then minced. The minced cartilage was digested with 2.5% of trypsin (Sigma Aldrich, Milan, Italy) at 37 °C for 10 min and then with 2 mg/mL of collagenase (Sigma Aldrich) for 16 h at 37 °C. Cells were then collected by using cell strainer 70 nm (EuroClone,), washed and placed in the culture medium consisting of Dulbecco’s Modified Eagle Medium (DMEM) supplemented with 10% fetal bovine serum (FBS), 100 U/mL penicillin, 100 μg/mL streptomycin in a humidified 5% CO_2_ atmosphere at 37 °C. The medium was changed every 48 h, until cells were split at 90% of confluence. All experiments were conducted using cells at two passages of subculture to minimize alterations in phenotypic drift associated with increased subculturing.

#### 2.1.2. PRP Preparation

PRP was prepared from whole blood using the double centrifuge method reported by Tognoloni et al. (2023) [26]. Blood was collected from two healthy horses by jugular venipuncture in acid citrate-dextrose (ACD) solution. Blood underwent two centrifugation steps, the first at 200× *g* for 20 min at 25 °C and the second at 1800× *g* for 10 min at 25 °C. The platelet pellet was then re-suspended in a 1 mL volume of platelet-poor plasma to obtain a final platelet concentration of 1 × 10^6^ platelets/μL; platelets counts were determined with a hemocytometer (EosBIO, Cervarese Santa Croce, Italy). The leukocyte concentration in the PRP preparations was notably low: 0.421 ± 0.1 × 10^3^/µL in the in vitro study and 0.39 ± 0.29 × 10^3^/µL in the in vivo study.

#### 2.1.3. Cell Viability Analysis

Cell viability was assessed using the 3-(4,5-dimethylthiazol-2-yl)-2,5-diphenyltetrazolium bromide (MTT) assay based on the conversion of MTT into a purple-colored formazan products by viable cells. Briefly, cells at density of 10 × 10^3^ cells/well were cultured in 96-well plates for 24 h in medium supplemented with 10% FBS. After a washing step in PBS, chondrocyte cells were exposed to 0.25, 0.5, 1, 2 and 4 mg/mL TA (Kenacort, Bristol Mayers Squibb, Rome, Italy) for 48 h in presence or in absence of PRP. Control cells were cultured in complete medium alone. After treatments, medium MTT solution (0.5 mg/mL) was added. After 2 h of incubation, the reaction was stopped by adding DMSO, which acts also for solubilizing formazan crystals. The absorbance was measured at 570 nm using a multiplate spectrophotometer (Infinite^®^ 200 Pro-Tecan, Männedorf, Switzerland). The experiments, conducted in triplicate, yielded mean values and standard deviation (SD) from four independent trials. Viability was expressed as percentage of ratio between optical density (OD) of treated cells and OD of control cells.

### 2.2. In Vivo Study

#### 2.2.1. Cases Selection

Patients included in this study were stabled at the Jockey Club of Turkey, Ankara Hippodrome, Turkey. The inclusion criteria were Thoroughbred racehorses active in racing and training, aged between 2 and 5 years old, who meet at least two of the following criteria: unilateral or bilateral MCP joint effusion, pain on passive flexion of the MCP joint, lameness localized to the MCP joint by clinical examination with or without intra-articular diagnostic analgesia. Additionally, radiographic findings consistent with OA of the MCP joint were part of the inclusion criteria. Horses were evaluated by two equine veterinarians, each with over 15 years of experience in the racing industry. All horses underwent a clinical and radiographic examination of the MCP joints. The static examination consisted of the evaluation of joint effusion and pain at passive flexion. In addition, the evaluation of painful response to passive flexion and joint effusion were graded using a four-point scale ranging from none to severe (0 = none, 1 = mild, 2 = moderate, 3 = severe). The dynamic examination included observing the horses trot in a straight line on hard surfaces. Lameness was graded from 0 to 5 using the modified AAEP grading scale [27]. The radiographic examination varied among the cases; however, at least the latero-medial, dorso 15° proximal-palmarodistal, dorso 45° proximolateral-palmarodistomedial oblique, dorso 45° proximomedial-palmarodistolateral oblique, flexed lateromedial and flexed dorsopalmar projections of the MCP joint were available [28]. Radiographs were assessed by the jockey club veterinarians and the presence of the following radiographic findings were recorded: periarticular osteophytes, capsular enthesophytes, subchondral bone sclerosis/lysis of the proximal phalanx and/or the metacarpal condyle and loss of joint space [28]. Exclusion criteria were horses with bilateral lameness and lameness graded as 4 and 5 on the AAEP grading scale, any type of fracture of the proximal phalanx and of the distal condyle of the metacarpus and any horse that was treated with any IA injection or other systematic anti-inflammatory therapy within four weeks before inclusion in the study. A total number of 53 MCP joints from 32 horses were included in this study, ranging between 2 and 5 years with a mean ± SD age of 2.7 ± 0.9 years.

#### 2.2.2. Treatments

**Figure 1:**
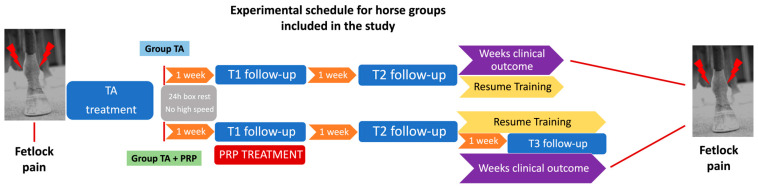
Flowchart of the experimental schedules for horse groups (group TA and group TA+PRP) included in the study.

The horses included in the study were randomly divided into two groups (Figure 1). The randomization was performed with a coin-flip randomization technique.

Both groups received one single intra-articular injection of 4 mg (2 mg/mL) of TA (Sinakort-A, Ibrahim Etem, Istanbul, Turkey) in the affected MCP joint/s.

The group TA+PRP received one single intra-articular injection of PRP one week after TA. Each joint received 1 ml of a platelet concentration of 1 × 10^6^ PLT/μL. They underwent 24 h of box rest and avoided high speed exercise for at least one week. All horses returned progressively to full training within 2–4 weeks. Follow-up clinical examinations were performed by the same clinicians for all time points. Effusion score, passive flexion score and lameness evaluations were recorded at one week (T1) and two weeks (T2) after TA administration in both groups (Figure 1) and at two weeks after PRP administration (T3) in group TA+PRP. In addition, the clinical outcome was evaluated recording the weeks between T2 and the return of the horse to the Jockey Club Hospital with the same complaint (i.e., MCP joint disease) including joint effusion, pain at flexion or lameness. Horses were monitored to check for any occurrence of adverse effects after treatments.

### 2.3. Statistical Analyses

The statistical analyses were performed using the statistical software JASP (version 0.18.1, Jasp Team, Amsterdam, The Netherlands). The quantitative data are expressed as mean ± SD or median and range, as appropriate; nominal data are expressed as prevalence and percentage. For numerical data (age, weeks of the clinical outcome), homoscedasticity of the variables was tested for normality using Shapiro–Wilk test and homogeneity of the variance with Levene test. Cell viability of the in vitro study was evaluated using one-way analysis of variance (ANOVA) and post-hoc Tukey test for multiple comparisons. For the in vivo study, descriptive statistics was applied for age, sex, limb affected (right forelimb or left forelimb), lameness score (baseline, T1, T2, T3), effusion score (baseline, T1, T2, T3), flexion score (baseline, T1, T2, T3) and clinical outcome (weeks). Differences in age and weeks of the clinical outcome between group TA and group TA+PRP were tested using the unpaired Student’s t-test or Mann–Whitney U test, as appropriate. Differences in sex and limb affected between the two groups were tested with the Chi-squared test. Two-way Kruskal–Wallis test with Dunn’s post-hoc correction for multiple comparisons were used to test for differences between the groups and time points (baseline, T1, T2, T3) for effusion score, passive flexion score and lameness score. All *p*-values less than 0.05 were considered significant.

## 3. Results

### 3.1. In Vitro Study

#### Effect of PRP on Chondrocyte Culture

**Figure 2:**
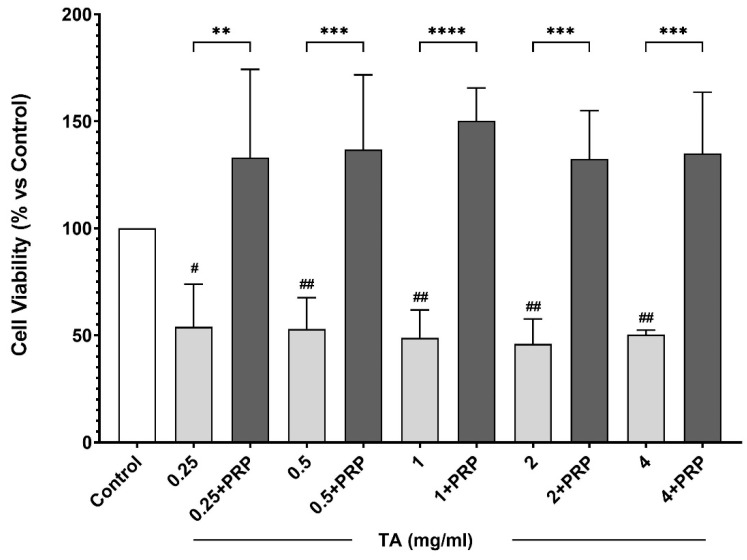
Cell viability after the addition of platelet-rich plasma (PRP) to the triamcinolone acetonide (TA) after 24 h at the treatment dose 0, 0.25, 0.50, 1.0, 2.0 and 4.0 mg/mL, as compared with triamcinolone acetonide alone. Data are the mean ± SD of four independent experiments performed in triplicates. ** *p* < 0.01; *** *p* < 0.001; **** *p* < 0.0001; # *p* < 0.05; ## *p* < 0.001 vs. control (CTRL).

Cell viability assessed by (MTT) test after 24 h of treatment with different concentrations of TA with or without PRP are shown in Figure 2. Exclusive use of TA led to a roughly 50% decrease in cell viability, beginning at the minimum dosage of 0.25 mg/mL (*p* < 0.05) compared to the control and maintaining a gradual decline as the concentration of TA increased to 4 mg/mL dosage vs. control (*p* < 0.01). In contrast, the combined use of TA with PRP demonstrated a protective effect on cellular viability, reaching average values around 130–140% for all the dosages and showing marked differences compared to TA treatment.

### 3.2. In Vivo Study

Among the 32 horses that were included in this study, there were 13 females and 19 intact males. Thirty-one joints (right forelimb = 17; left forelimb = 14) from 18 horses were in group TA and twenty-two joints (right forelimb = 12; left forelimb = 10) from 14 horses were in group TA+PRP. In the group TA, from a total of 18 horses, 13 were bilateral and five unilateral affected, and in the group TA+PRP, from a total of 14 horses, 8 were bilateral and six unilateral affected. There were no differences in age (*p* = 0.06), sex (*p* = 0.33), limb affected (*p* = 0.98) between the group TA and group TA+PRP.

In group TA, 24 out of 31 MCP joints showed effusion; a total of 29 joints had positive responses to the passive flexion and six forelimbs were lame in the TA group. In group TA+PRP, 19 out of 22 MCP joints showed effusion; a total of 21 joints had a positive response to the passive flexion and eight forelimbs were lame. The details of effusion score, passive flexion score and lameness score at each time point in the two groups are summarized in Table 1.

**Figure 3:**
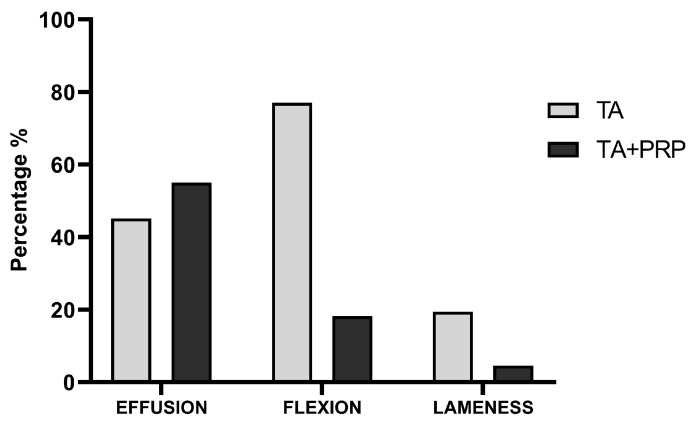
Reasons for re-admission to the hospital following the treatments in the TA and TA+PRP groups. The chart demonstrates the frequency of recurring clinical signs observed during re-admission to the hospital, revealing variations in effusion, flexion and lameness complaints between the TA and TA+PRP groups.

There were no significant differences in the effusion score between the groups at each time point (*p* = 0.77). For both groups, the effusion score at T1 (*p* < 0.001) was significantly lower than that at baseline; for group TA+PRP, T2 and T3 also were significantly lower than that at baseline (*p* < 0.001). There was no significant difference between T1 and T2 (*p* = 0.1). There were significant differences in the flexion scores between the groups (*p* = 0.04) and the time points (*p* < 0.001). There was a significant reduction in the passive flexion score in both groups between baseline and T1 (*p* < 0.001) and T2 (*p* < 0.001); however, the passive flexion score in the group TA+PRP at T2 and T3 were significantly lower compared to that of the group TA at T2 (*p* = 0.007 and *p* < 0.001, respectively). There were no significant differences in the lameness score between the groups at each time point (*p* = 0.73), but a significant interaction between group*time was found. Only in group TA+PRP, the lameness score significantly decreased at T2 (*p* = 0.002), T3 (*p* = 0.002), compared to the baseline. Finally, there was a significant difference (*p* < 0.001) in the outcome weeks between the two groups. Horses in which the MCP joint/s was/were treated with TA (group TA) were re-admitted early (median: 4 weeks; range: 4–5 weeks) to the Jockey Club Hospital compared to horses in which the MCP joint/s was/were treated with TA and PRP (group TA+PRP) (median: 7 weeks; range: 6–8 weeks). The causes of re-admission to the hospital are depicted in Figure 3. In the group TA+PRP, the cause of readmission was effusion in 18 (82%) joints, pain on flexion was noted in 4 joints (18%) and lameness was observed in 1 out of 22 horses (5%). In the TA group, the cause of re-admission was effusion in 24 (77%) joints, pain on flexion in 14 (45%) and lameness was observed in six horses (19%).

## 4. Discussion

Intra-articular corticosteroids are often used as a first-line treatment for OA affecting equine athletes because they can improve range of motion and effusion of the MCP joints and relieve pain [3]. However, the use of IA corticosteroid is a questionable issue, because they can result in changes of chondrocyte metabolism, cellular toxicity, mitochondrial dysfunction, reactive oxygen species increase and cell death [29,30]. In this study, the effect of combining TA and PRP has been investigated and compared to the effects of TA alone, in vivo and in vitro. Firstly, the potential detrimental effects of TA on equine chondrocytes have been demonstrated in vitro. Indeed, we observed a significant decrease in the viability of equine chondrocytes induced by TA (0.25–4 mg/mL), consistent with those demonstrated by other authors exposing human chondrocytes to similar [31] and higher concentrations [32,33]. Similar effects have been also observed in rabbit chondrocytes [10,34,35] and canine chondrocytes [29,36].

In contrast, the combined use of TA and PRP led to an increase in cell viability, indicating a clear protective effect of PRP on the cytotoxicity exerted by TA in vitro. These findings support the results obtained on human chondrocytes exposed to other corticosteroids [19,21]. Additionally, several studies have reported the cytoprotective effects of PRP against the toxicity of various drugs on different fibroblast cell types, including chondrocytes [23,37,38,39,40]. For example, PRP increased the cell viability and decreased apoptosis of human rotator cuff tear cells exposed to TA [18,41]. By speculating on our results, it is plausible to hypothesize that PRP can protect chondrocytes by counteracting the pro-oxidant effects of TA. Indeed, it has been reported that TA significantly increases the levels of oxidized glutathione, leading to oxidative stress in human chondrocytes [42]. In contrast, PRP appears to enhance the antioxidant cellular response via the NRF2 pathway [26,43].

The results of our in vivo study supported the role of the IA administration of TA+ PRP as able to improve the clinical signs in horses with positive flexion test of the MCP joint and/or lameness due to its anti-inflammatory activity [44], as well as the synergistic effect of PRP when combined with other drugs [44,45]. Moreover, these effects could be also related to the ability of PRP in promoting chondrocyte proliferation and cartilage matrix secretion and in stimulating cartilage repair [46,47]. It has been reported that PRP exerts beneficial effects on joint cartilage, synovium, tendon and overall healing processes [48,49,50]. Specifically, anabolic effects of PRP have been observed in porcine chondrocyte cultures, highlighting its regenerative potential in cartilage tissue [51,52].

Synovitis, trauma or injury can lead to activation of the mechanoreceptors [53,54], which in turn can stimulate an inflammatory response with the release of pro-inflammatory cytokines and degradative enzymes (IL-1B and TNF-alpha and MMPs). This further increases the amount of joint swelling and further activates nociceptors, perpetuating pain. All these events cause additional osteochondral damage and cartilage degeneration in OA [54]. Acute synovitis is considered the most common problem in equine high-motion joints, which includes the MCP joint, contributing to the degradative mechanism of the articular cartilage [55]. For these reasons, it was not surprising that the effusion score in this study group improved after both treatments. Both groups received TA, which is a potent anti-inflammatory drug inhibiting the inflammatory process at all levels [56,57]. However, the effusion score reduced at T1 compared to the baseline and remained significantly lower at T2 and T3 in group TA+PRP; in contrast, effusion score in group TA returned not significantly different compared to the baseline at T2. Overall, there were no differences in the effusion score between the two groups.

A painful response to passive flexion of the joint and lameness are well known clinical signs of pain in horses and human beings [58,59]. In our clinical study, a significant reduction in the passive flexion score was demonstrated in both groups. However, in contrast to group TA, the group TA+PRP maintained a lower flexion score compared to the baseline at T1, T2 and T3. The group TA+PRP had a longer effect in maintaining lower flexion scores, while group TA showed a worsening of flexion scores at T2 compared to T1, even if the score remained lower to that of the baseline. Indirectly, this difference demonstrated a shorter analgesic effect in horses treated only with TA compared to that treated by TA followed by PRP. The lameness score was another clinical variable considered. Similarly, despite the low number of horses presenting lameness, a significant decrease in the lameness score was recorded in group TA+PRP at T2 and T3, which was not the case of group TA. These findings may explain the favorable effect of TA+PRP group on lameness score as PRP products were shown to be effective in relieving clinical signs of OA [59,60].

In support of the beneficial use of PRP after TA, authors would highlight that those horses treated with TA alone returned to the hospital in a shorter time (4.4 weeks on average), compared with horses treated with TA and PRP (7.1 weeks on average). This may be attributed to the therapeutic additional effects of PRP, offering a promising approach to reducing the adverse outcomes associated with corticosteroid use in joint treatments [21,61]. The short duration of improvement observed in this study may be related to several contributing factors influencing the recurrence of clinical signs. In the literature, the most common doses of TA administered by equine practitioners range from 5 to 10 mg, with a therapeutic duration rarely exceeding 4 to 6 weeks [62,63,64,65]. In our study, a dose of 4 mg per joint was used, and the re-admission of the horses in the TA group at 4.4 weeks aligns with previous studies [62,63,64,65]. Other factors include training strategies, the overall management of the horses, as well as the experience and quality of the staff and riders. Another important point might be the training track surfaces. Thus, horses may be predisposed to recurrence of clinical signs if trained at high intensity on surfaces to which they are not accustomed [65,66]. Additionally, horses with high athletic demands may experience greater stress on their joints, leading to faster recurrence of issues despite treatment.

Moreover, the fact that the flexion score of the horses in the group TA+PRP did not worsen in the weeks following the treatment and that the owners of these horses did not complain in training showed that PRP application had a positive effect on pain and longer suppression of the clinical signs. In human studies, it has been demonstrated that PRP improves joint and tissue function, relieves pain and results in favorable clinical outcomes [67,68]. However, various studies have shown that PRP is a more suitable method for use in humans than horses. Interestingly, in our study, no adverse effects of IA administration of PRP were recorded, even though it has been reported that this treatment may induce a reaction or transient synovitis [13]. The side effects of the IA administration of PRP are likely to involve the preparation and standardization protocols of PRP and in particular, the leucocyte concentration [69,70]. Overall, the authors suggest that the PRP has a safe profile when used IA and might have a pivotal role in disease progression due to its ability to protect chondrocytes by reducing the adverse effect of TA.

There are some limitations in this study. The first limitation is a lack of the use of diagnostic analgesia in some horses to determine if the presence of lameness was due to pain in the MCP joint. From the clinical perspective, it is fair to suggest that the distal limb flexion test is sensitive to examining the MCP joint, but it may be less significant for tissues distal to the joint. Two researchers suggest that the MCP joint is the primary contributor to a positive flexion test [71,72], but clinical signs and radiographic evidence should also be evaluated for a thorough evaluation. For this reason, to avoid bias, more than one criterion was used as inclusion criteria in this study group. The second limitation is that synovial samples were not analyzed to assess changes in synovial biomarkers. As a result, this hypothesis remains to be validated experimentally, and it will be the aim of our next study. The third limitation is that in vitro, PRP was added concomitantly with TA-treated chondrocytes, whereas in vivo, PRP was administered one week after TA. This timing also may have influenced the clinical results. However, the in vivo study was conducted on horses with OA clinical signs, while the in vitro study used healthy chondrocytes treated with TA. Additionally, tracking the effects of TA for one week on chondrocytes in vitro is challenging, as we observed cytotoxicity even at lower doses. Administering TA prior to PRP allowed us to verify if PRP treatment had additional effects in joints with reduced inflammation.

Finally, horses with similar pathologies may exhibit individual responses to treatment. The therapeutic effects may also be influenced by the horses’ working discipline and post-treatment exercise protocols. This individual response was the reason for separate administration between TA and PRP.

## 5. Conclusions

There are many studies in the literature about the use of corticosteroids and PRP, but in equids the use of combined TA and PRP has limited study. The studies evaluating the cytotoxic effects of TA on equine chondrocytes are also limited and controversial. This study is the first to investigate in vitro the potential harmful effects of this corticosteroid on equine cartilage cells and the possible protective effect of PRP when administered together with this drug. The results of the in vivo study suggest a promising strategy to alleviate any adverse effect on chondrocyte viability after the corticosteroid administration, highlighting the potential for mid-term pain relief and reducing lameness through a strategically timed PRP injection. Indirectly, these results could indicate that PRP could elicit a proliferative effect on chondrocytes, albeit minor, despite the presence of TA. Further clinical trials are crucial for a comprehensive evaluation of the therapeutic potential and safety profile associated with the integration of PRP with TA in the treatment of osteoarthritis in equine athletes. Multiple PRP injections are likely to lead to a better clinical outcome than a single injection. Comparing multiple PRP injections after a single dose of TA versus a single dose of TA alone may reveal changes in outcome [73,74,75]. However, this needs further investigations, which might also provide a better understanding of the beneficial effect of PRP injections in the long term.

## Figures and Tables

**Table 1:** Median and range of effusion score, passive flexion score, lameness score, weeks of the follow-up and frequencies of adverse effects obtained in group TA and group TA+PRP.

Groups	Baseline	T1		T2	T3	*p*-Value *
	Effusion score (median; range)					
**Group TA (*n* = 31)**	1; 0–3	0; 0–1 ^a^		0; 0–2	na	<0.001
**Group TA+PRP (*n* = 22)**	2; 0–3	0; 0–1 ^a^		0; 0–1 ^a^	1; 0–1 ^a^	<0.001
***p*-value** ^#^	1.00	1.00		0.87		
	Passive flexion score (median; range)					
**Group TA (*n* = 31)**	2; 0–3	0; 0–2 ^a^		**1; 0**–**2 ^a,b^**	na	<0.03
**Group TA+PRP (*n* = 22)**	2; 0–3	0; 0–2 ^a^		**0; 0**–**2 ^a^**	**0; 0**–**1 ^a,c^**	<0.001
***p*-value** ^#^	1.00	1.00		**0.007**	**<0.001**	
	Lameness score (median; range)					
**Group TA (*n* = 31)**	0; 0–2	0; 0–1		0; 0–1	na	1.00
**Group TA+PRP (*n* = 22)**	0; 0–3	0; 0–1		0; 0–1 ^a^	0; 0–1 ^a^	0.002
***p*** **-value ^#^**	0.36	1.00		1.00		
	Weeks of follow-up (median; range)					
**Group TA (*n* = 31)**	**4; 4–5**					na
**Group TA+PRP (*n* = 22)**	**7; 6–8**					na
***p*** **-value ^#^**	**<0.001**					
	Adverse Effects (prevalence; %)					
	**No**		**Yes**			
**Group TA (*n* = 31)**	31 (100%)		0 (0%)			
**Group TA+PRP (*n* = 22)**	22 (100%)		0 (0%)			
***p*** **-value ^#^**	na					

* *p*-value of multiple comparison for time; ^#^ *p*-value of the multiple comparison for time. Bold defined significant differences between group TA and group TA+PRP; ^a^ defined significant difference between T_X_ and baseline (*p* < 0.05); ^b^ defined significant difference between T_X_ and T1 (*p* < 0.05); ^c^ defined significant difference between T2 of Group TA and T3 of Group TA+PRP (*p* < 0.05); na = not applicable.

## Data Availability

The data presented in this study are available on request from the corresponding author.
